# Efficacy of Topical Hydrocortisone in Combination with Topical Ciclosporin A for the Treatment of Dry Eye Disease in Patients with Sjögren Syndrome

**DOI:** 10.1155/2021/7584370

**Published:** 2021-11-30

**Authors:** Klemens Fondi, Kata Miháltz, Pia Veronika Vécsei-Marlovits

**Affiliations:** ^1^Department of Ophthalmology, Hietzing Hospital, Vienna, Austria; ^2^Karl Landsteiner Institute of Process Optimization and Quality Management in Cataract Surgery, Vienna, Austria

## Abstract

**Introduction:**

The aim of this randomized, observer-masked, parallel group study was to evaluate the short-term and long-term effects of topical hydrocortisone administered in addition to topical ciclosporin A for the first 2 weeks of the treatment in patients with dry eye disease associated with Sjögren syndrome.

**Materials and Methods:**

24 eyes of 12 patients with severe dry eye disease associated with Sjögren syndrome were included in this study. Both eyes of all patients were treated with preservative-free Ciclosporin A eye drops once daily for 6 months. Additionally, one eye of each patient received hydrocortisone eye drops three times daily for the first two weeks of treatment. The study parameters were assessed before treatment, after 2 weeks, and after 6 months of treatment.

**Results:**

Tear BUT and corneal fluorescein Oxford staining grade showed significant differences with respect to the baseline when treated with ciclosporin A and hydrocortisone (CsA + Hc) and a nonsignificant increase when treated with ciclosporin A (CsA) alone. After 6 months of treatment, significant increases of tear BUT and corneal Fluorescein Oxford staining grade compared to baseline could be observed in both treatment groups. Aberrometry measurements showed significantly increased optical image quality after 6 months in the CsA + Hc group, while no significant changes could be detected in the eyes treated with CsA alone. However, no significant differences between the two treatment groups could be detected. *Discussion*. This study indicates that hydrocortisone combined with ciclosporin A therapy may provide fast improvement of clinical symptoms and could have long-term positive effects on the optical image quality in severe DED patients with Sjögren syndrome.

## 1. Introduction

Dry eye disease (DED), also called keratoconjunctivitis sicca, is a multifactorial disorder of the tear film and ocular surface, resulting in tear film instability with potential damage to the ocular surface [1]. The symptoms of DED can vary from slight ocular discomfort in mild cases to ocular pain and impairing visual disturbance in severe cases [2]. Since DED is a multifactorial disorder, a careful investigation of the possible causes and risk factors is essential. The major risk factors for the development of DED are age, female sex, contact lens wear, computer use, environmental conditions, systemic medications, and autoimmune disorders [3]. An important cause for severe DED is Sjögren syndrome. Sjögren syndrome is an autoimmune disease affecting the function of the lacrimal and salivary glands amongst other organs. Sjögren syndrome can occur independently of other diseases as primary Sjögren syndrome or can be associated with other autoimmune diseases like rheumatoid arthritis or systemic lupus erythematosus as secondary Sjögren syndrome [4]. The diagnostics of this disease include both objective clinical signs and subjective symptoms, the intensity of which often does not correlate with one another [5]. The most frequently used and most meaningful diagnostic methods are the tear film breakup time (BUT), ocular surface staining with fluorescein and lissamine green, Schirmer test, the assessment of the Meibomian glands, and the assessment of subjective complaints, such as the most commonly used ocular surface disease index (OSDI) questionnaire [6].

A further frequently observed and potentially impairing issue in DED patients is the reduction of optical quality [7]. Visual disturbances in patients with DED are caused by the irregularities of the tear film and the ocular surface, causing considerable subjective vision impairment [8]. These visual impairments are often difficult to quantify with conventional visual acuity testing. Hence, particularly in patients with DED, wavefront aberrometry can be used to objectively determine the extent of optical quality disturbances and assess the effect of treatment [9,10]. Patients with mild or moderate DED can be treated with the topical lubricants and stimulation of the Meibomian glands to restore the stability of the tear film and reduce ocular discomfort [11]. To treat severe DED, especially when associated with Sjögren syndrome, it is often necessary to use topical antiinflammatory medication in addition to lubricating eye drops to break the vicious circle of immune response and damage to the ocular surface [12]. Topical corticosteroids have been shown to reduce the signs and symptoms of DED associated with Sjögren syndrome with the side effects of intraocular pressure elevation, cataract formation, and opportunistic infections, resulting in only short-term treatment durations [13,14]. In contrast to other corticosteroids, such as dexamethasone and prednisolone, hydrocortisone has been observed to have limited penetration of the cornea, triggering fewer side effects and higher effectiveness on the ocular surface [15,16].

Furthermore, topical ciclosporin A is an effective treatment option for severe DED with fewer side effects than corticosteroids, resulting in the recommended treatment duration of up to 6 months [4,17]. The onset of symptom relief observed by patients treated with ciclosporin A has shown a latency, leading to possible issues with treatment adherence in these patients [18,19]. As a result, topical corticosteroids are often used as pretreatment or, in addition, for the first weeks of treatment with topical ciclosporin A [20,21].

The aim of this study was to evaluate the short-term and long-term effects of topical hydrocortisone administered in addition to topical ciclosporin A for the first 2 weeks of the treatment in patients with DED associated with Sjögren syndrome.

## 2. Materials and Methods

The study was performed in accordance with the Declaration of Helsinki and the Good Clinical Practice (GCP) guidelines of the World Health Organization. The study protocol was approved by the local Ethics Committee of the Medical University of Vienna. All subjects gave written informed consent after detailed personal explanation. Twelve male and female patients with severe DED associated with primary or secondary Sjögren syndrome defined were included in this study. Sjögren syndrome was diagnosed in cooperation with the hospital's Rheumatology Department according to the American-European Consensus Group criteria, including the subjective criteria comprising ocular symptoms and oral symptoms, as well as the objective criteria comprising the ocular signs, histopathology, salivary gland involvement, and autoantibodies [22]. All patients had to have a stable course of the disease with unchanged treatment for 6 months before their inclusion into the study. The diagnosis of severe DED was defined by the staining of the cornea ≥ grade III according to the Oxford scale and an OSDI value ≥ 25. The patient's age had to be between 18 and 90 years, and pregnancy was excluded with a pregnancy test in patients of childbearing potential. The patients had to be under treatment with lubricating eye drops for at least 6 months without a satisfying improvement of ocular symptoms. The exclusion criteria were eye surgery in the past 6 months, regular use of eye drops with the exception of tear substitutes, the use of eye drops containing ciclosporin A or corticosteroids in the last 6 months, and simultaneous participation in another study.

### 2.1. Study Design and Treatment Regimen

This study was performed in a randomized, observer-masked design. Both eyes of all patients included in this study were treated with preservative-free ciclosporin A 0.1% eye drops (Ikervis, Santen Pharmaceutical, Osaka, Japan) once daily in the evening for 6 months. One eye of each patient received, additionally, hydrocortisone 0.335% (Softacort, Laboratoires Thea, Clemont-Ferrand, France) eye drops three times daily for the first two weeks of treatment. The eye receiving additional hydrocortisone eye drops was determined by randomization. In addition, all patients included in this study received preservative-free Artelac complete (Bausch & Lomb GmbH, Berlin, Germany) eye drops 5 times per day, containing sodium hyaluronate, carbomer, and medium chain triglycerides.

### 2.2. Examination Methods

Tear breakup time: tear breakup time (BUT) was measured according to the guidelines described in the report of the TFOS International Dry Eye Workshop [6]. One drop of Minims-Fluorescein sodium 2.0% eye drops (Bausch & Lomb, Rochester, USA) was administered in the conjunctival sac. The patients were instructed to blink naturally several times to distribute the fluorescein. Thereafter, the patients were asked to look straight without blinking until instructed otherwise. The slit-lamp illumination intensity was kept constant with the cobalt blue light, and the magnification was set at 10x. The time from the last complete blink and the first appearance of an unstained spot was recorded. The tear breakup time was measured three times and the average value was taken for analysis.

Corneal fluorescein staining: Minims-Fluorescein sodium 2.0% eye drops (Bausch & Lomb, Rochester, USA) were applied to detect corneal epithelial defects using the cobalt blue light. A yellow filter was used to enhance the observation of corneal staining. Oxford grading scale was used for quantifying the corneal epithelial damage [6].

Conjunctival lissamine staining: lissamine green ophthalmic strips (Optitech Eyecare, Prayagraj, India) were moistened with a drop of sterile saline and shortly placed in the lower fornix of the eye to stain the ocular surface. Oxford grading scale was used for quantifying conjunctival damage [6].

Schirmer I test: Schirmer I test without anaesthesia was performed according to the guidelines published in the report of the TFOS International Dry Eye Workshop [6]. Schirmer paper strips were inserted in the eye between the middle and outer third of the lower lid margin, and the patients were asked to close their eyes. After 5 minutes, the wetting of the Schirmer paper was measured in millimeters.

Intraocular pressure: The intraocular pressure (IOP) was measured with a Goldmann applanation tonometer attached to a slit lamp. Before the measurement, one drop of oxybuprocaine hydrochloride (4.0 mg/mL) combined with sodium fluorescein (0.8 mg/mL) was applied. IOP measurements were performed at the end of each study visit to avoid interference with other examinations.

Ocular surface disease index (OSDI): the symptoms of DED were assessed using the OSDI questionnaire. The patients were asked to fill out the questionnaire by grading the frequency of specific DED-related symptoms and their impact on vision-related activities during the last week. The results are reported on a scale from 0 to 100, where 0 means no symptoms and 100 means maximal symptoms of DED.

The assessment of ocular discomfort using Visual Analogue Scale (VAS): to assess ocular discomfort separately for each eye, Visual Analogue Scale (VAS) was used. The patients were asked to mark the degree of their symptoms on a line of 100 mm length, on which 0 means no symptoms and 100 means the worst possible symptoms. The following symptoms were evaluated using VAS: foreign body sensation, burning, itching, pain, sticky sensation, blurred vision, sensitivity to light, and the frequency of dry sensation. The mean of all symptom scorings was calculated to assess the degree of ocular discomfort, separately, for each eye.

Meibography measurements: for meibography measurements, the cobra fundus camera system (Bon Optic, Lübeck, Germany) was used, which includes a high-resolution infrared camera and the Phoenix digital analysis software. After everting the lower eyelid and taking infrared images, the software calculates the ratio of the existing Meibomian gland area relative to the total analysis area. Three consecutive images from the everted lower eyelid were captured and the average of 3 values was used for analysis.

Aberrometry measurements: corneal optical aberrations were measured using the iTrace Visual Function Analyzer (Tracey Technologies, Houson, USA). Aberrometry measurements with this device comprise keratometry, autorefraction, pupil diameter, topography, and wavefront aberrations, simultaneously, on the same axis. The measurements were performed with the patient focusing on a distant target at a fixed entrance pupil scan size of 4.0 mm and 3 to 5 seconds after blinking. The measurements were repeated at least 3 times. The scan with the best measurement quality was chosen for analysis. The optical quality was described by the modulation transfer function (MTF) and the Strehl ratio. Corneal aberrations alone were included in our analysis since DED and the treatments used in this study are not expected to cause changes in the inner eye structures.

Data and statistical analysis: SPSS 25 software (SPSS Inc, Chicago, IL) was used for statistical analysis. All data are presented as mean with standard deviation. To compare parameters between the two treatment groups, a student's *t* test for independent samples was used for interval scaled data. The Mann–Whitney U test was used for ordinal scaled data. To evaluate the outcomes among the baseline, after 2 weeks of treatment, and after 6 months of treatment, a *t* test for the paired samples was used for the interval scaled data and a Wilcoxon signed-rank test was used for the ordinal scaled data. *P* ≤ 0.05 was considered statistically significant.

## 3. Results

In this study, 24 eyes of 12 patients with Sjögren syndrome were included. The demographic data of the study population are shown in Table 1. Two of the patients were male and ten were female with a mean age of 62.3 ± 18.5 years. Three patients had been diagnosed with primary Sjögren syndrome, while nine of the included patients had secondary Sjögren syndrome associated with rheumatoid arthritis or systemic lupus erythematosus. All patients finished the study according to the protocol.

The study parameters at the baseline compared between the two treatment regimens are shown in Table 2. At the baseline, no statistically significant differences between the two treatment groups were found.

**Figure 1:**
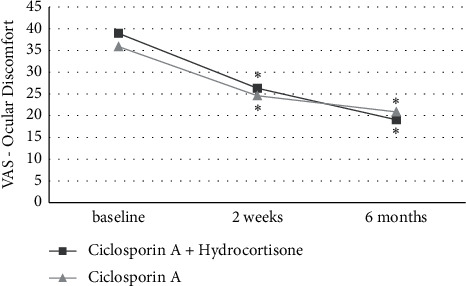
Visual Analogue Scale (VAS) of subjective ocular discomfort in both treatment groups at baseline, after 2 weeks, and after 6 months of treatment. Significant differences to baseline are marked with ^*∗*^.

An overview of all study parameters at the baseline after 2 weeks of treatment and after 6 months of treatment is given in Table 3 separately for the two treatment regimens. In contrast to the other study parameters, the Ocular Surface Disease Index (OSDI) could not be assessed separately per eye and treatment regimen. However, it shows a significant decrease of the general dry eye symptoms compared to the baseline after two weeks of treatment (−13.9 ± 11.8, *P* = 0.002) and a further decrease after 6 months of treatment (−27.8 ± 15.2, *P* < 0.001). As shown in Figure 1, the Visual Analogue Scale decreased significantly in both treatment groups compared to the baseline after 2 weeks (−12.6 ± 7.8, *P* < 0.001 vs. −11.3 ± 9.4, *P* = 0.002) and after 6 months (−19.9 ± 11.3, *P* < 0.001 vs. −15.0 ± 12.1, *P* = 0.001) of treatment, but with larger decreases in the CsA + Hc group compared to the CsA treatment group.

**Figure 2:**
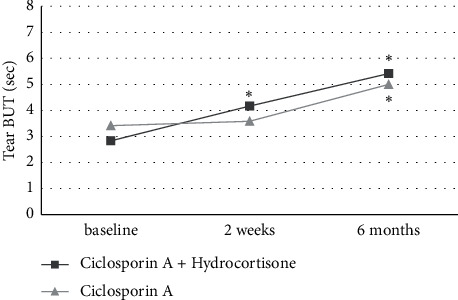
Tear breakup time (BUT) in both treatment groups at baseline, after 2 weeks, and after 6 months of treatment. Significant differences to baseline are marked with ^*∗*^.

**Figure 3:**
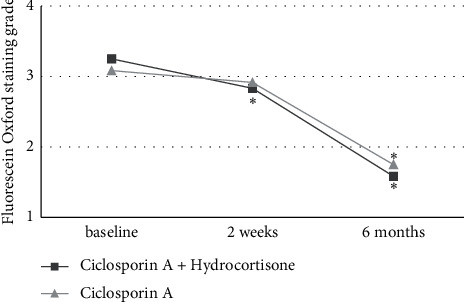
Fluorescein Oxford staining grade in both treatment groups at baseline, after 2 weeks, and after 6 months of treatment. Significant differences to baseline are marked with ^*∗*^.

**Figure 4:**
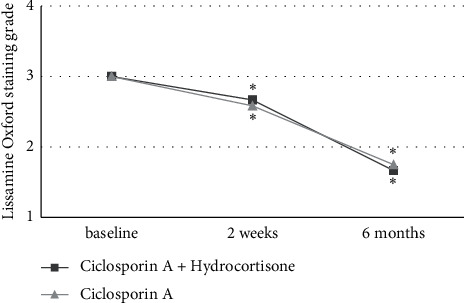
Lissamine Oxford staining grade in both treatment groups at baseline, after 2 weeks, and after 6 months of treatment. Significant differences to baseline are marked with ^*∗*^.

As depicted in Figure 2, tear BUT increased in both treatment groups after two weeks with significant differences with respect to the baseline only in the CsA + Hc group and a nonsignificant increase in the CsA group (+1.3 ± 1.3, *P* = 0.008 vs. +0.2 ± 1.7, *P* = 0.493). After 6 months of treatment, the significant increases of tear BUT compared to baseline could be observed in both treatment groups (+2.6 ± 1.5, *P* = 0.003 vs. +1.6 ± 1.2, *P* = 0.007). Similar results were seen in corneal Fluorescein Oxford staining grade, as shown in Figure 3. Significant differences to the baseline in corneal Fluorescein Oxford staining grade after two weeks of treatment only were found in the CsA + Hc group (−0.4 ± 0.5, *P* = 0.025 vs. −0.2 ± 0.6, *P* = 0.317), while after 6 months of treatment, significant differences to the baseline were seen in both treatment regimens (−1.7 ± 0.5, *P* = 0.001 vs. −1.3 ± 0.8, *P* = 0.004). The significant decreases of the conjunctival Lissamine Oxford staining grade in both groups compared to the baseline after 2 weeks (−0.3 ± 0.5, *P* = 0.046 vs. −0.4 ± 0.5, *P* = 0.025) and after 6 months of treatment (−1.3 ± 0.8, *P* = 0.002 vs. −1.3 ± 0.8, *P* = 0.002) are illustrated in Figure 4.

**Figure 5:**
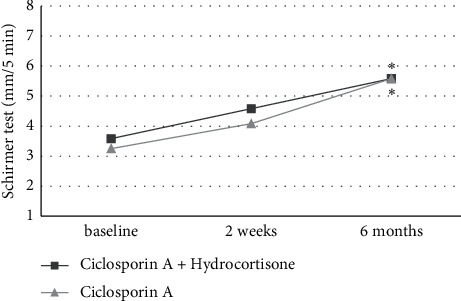
Schirmer test in both treatment groups at baseline, after 2 weeks, and after 6 months of treatment. Significant differences to baseline are marked with ^*∗*^.

The Schirmer test values showed in both treatment groups nonsignificant increases compared to baseline after 2 weeks (+1.0 ± 1.7, *P* = 0.086 vs. +0.8 ± 1.9, *P* = 0.057) and significant increases compared to the baseline after 6 months of treatment (+2.0 ± 3.0, *P* = 0.049 vs. +2.3 ± 2.9, *P* = 0.050), as shown in Figure 5. The Meibomian gland dropout rate did not show significant differences to baseline after 6 months of treatment (−1.3 ± 2.4, *P* = 0.094 vs. −1.4 ± 2.5, *P* = 0.070). Also nonsignificant changes of the intraocular pressure were found in either of the treatment groups after 2 weeks (+0.6 ± 2.9, *P* = 0.289 vs. +0.3 ± 2.2, *P* = 0.757) and after 6 months (+0.4 ± 2.8, *P* = 0.952 vs. +0.3 ± 2.8, *P* = 0.836).

**Figure 6:**
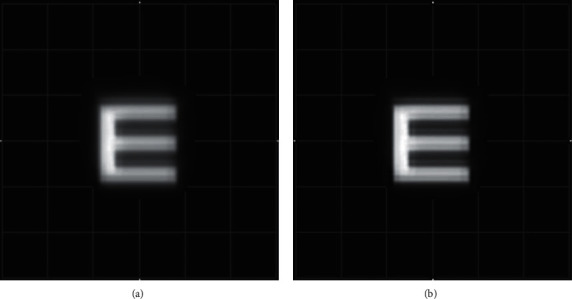
Simulated visual acuity images from the aberrometry measurements of a patient treated with topical ciclosporin A before (a) and after therapy (b).

**Figure 7:**
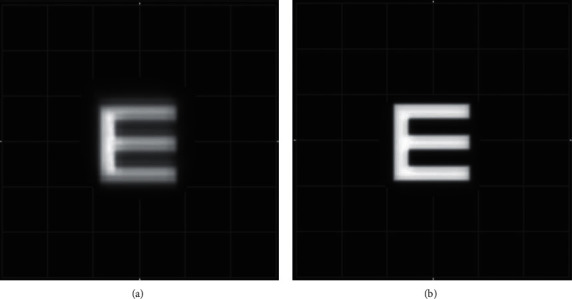
Simulated visual acuity images from the aberrometry measurements of a patient treated with topical ciclosporin A and hydrocortisone before (a) and after therapy (b).

Concerning optical quality parameters, both corneal MTF and corneal Strehl ratio showed significant changes in the CsA + Hc treatment group after 6 months (+0.12 ± 0.18, *P* = 0.037 and + 0.13 ± 0.19, *P* = 0.042), while nonsignificant changes of corneal MTF and corneal Strehl ratio were observed in the CsA treatment group after 6 months compared to baseline (+0.02 ± 0.01, *P* = 0.923 and + 0.01 ± 0.02, *P* = 0.945). The differences between the two treatment groups regarding optical image quality are demonstrated in Figures 6 and 7. Simulated visual acuity images resulting from aberrometry measurements of a patient in the CsA treatment group before and after the therapy are shown in Figure 6, while Figure 7 depicts the simulated visual acuity images of a patient in the CsA + Hc treatment group.

The *P* values of differences in all study parameters between the two treatment regimens are shown in Table 4. After 2 weeks and after 6 months of treatment, no statistically significant differences between the CsA + Hc group and the CsA group were found.

## 4. Discussion

Several studies have shown the effectiveness of topical ciclosporin A for the treatment of moderate-to-severe DED [18,23]. Inflammatory mechanisms are important factors in the pathophysiology of DED, especially when associated with Sjögren syndrome, which is why the antiinflammatory effects of ciclosporin A can be of use, especially in these cases [1,24]. Also, topical corticosteroids have potent and fast acting antiinflammatory effects with the potential of breaking the vicious circle between the immune response and damage to the ocular surface in DED [14]. The fast relief of symptoms and clinical improvement make corticosteroids an attractive therapeutic choice for the treatment of severe DED. While ciclosporin A has to be used for a longer period of time to take full effect, corticosteroids should not be administered long-term because of possible side effects, such as the elevation of intraocular pressure, cataract development, and opportunistic infections [25].

Since the effects of ciclosporin A have shown a latency until the effects on clinical signs and symptoms can be observed, topical corticosteroids are often used as pretreatment medicines or, additionally, for the first weeks of treatment [21,26]. Bjun et al. showed that topical ciclosporin A combined with topical methylprednisolone provided faster symptom relief and improvement of DED signs than ciclosporin A alone [20]. Singla et al. found similar benefits of treating moderate DED with loteprednol in addition to cclosporin A [26]. In contrast to other corticosteroids, hydrocortisone has shown low ocular penetration, resulting in a lower risk of side effects. It is available in a preservative-free formulation [15].

Therefore, this study had the aim to investigate the short-term and long-term effects of topical hydrocortisone administered in addition to topical ciclosporin A for the first 2 weeks of the treatment in patients with DED associated with Sjögren syndrome.

The results of the OSDI questionnaire show significantly lower values after 2 weeks of treatment and 6 months of treatment, indicating generally good effectiveness of the treatment regimen in terms of symptom reduction, independently, of the treatment group. Visual Analogue Scale results regarding ocular discomfort showed greater improvements in the CsA + Hc group compared to the CsA treatment group, indicating a slightly better short-term and long-term impact on the subjective DED symptoms of topical hydrocortisone in addition to topical ciclosporin A compared to the treatment with topical ciclosporin A alone.

Tear BUT and corneal Fluorescein Oxford staining grade showed a significant improvement compared to the baseline in the CsA + HC treatment regimen after 2 weeks of treatment, while nonsignificant changes were observed in the CsA group. This may indicate the short-term effectiveness of topical hydrocortisone in addition to topical ciclosporin A in improving clinical DED signs. These results are in accordance with the work of Byun et al. and Singla et al., evaluating the efficacy of topical methylprednisolone and loteprednol, respectively, in combination with ciclosporin A for the treatment of moderate-to-severe DED [20,26].

Conjunctival Lissamine Oxford staining grade significantly decreased in both treatment groups already after 2 weeks of treatment with further decreases after 6 months. This suggests the rapid effectiveness of topical ciclosporin A for improving the conjunctival ocular surface damage without the benefit of additional topical hydrocortisone. The amount of tear fluid production measured by the Schirmer test increased similarly in both treatment groups with nonsignificant changes after 2 weeks and significant changes after 6 months of treatment. This observation could be the result of the long-term antiinflammatory effects of ciclosporin A on the accessory lacrimal glands [27]. Byun et al. and Singla et al. found differences in the Schirmer test already after 2 weeks and 1 month of treatment, which could be explained by the less severe stages of DED in the populations of these studies.

The Meibomian gland dysfunction and dropout has been shown to be highly prevalent in Sjögren syndrome as a result of chronic inflammatory reactions of the ocular surface [28,29]. The present study also shows a high dropout rate of the Meibomian glands, which did not change after the antiinflammatory therapy of both treatment regimens. These results suggest that the atrophic Meibomian glands in Sjögren syndrome cannot be reactivated with topical antiinflammatory treatment. On the other side, the increased tear BUT indicates the stimulation of the remaining Meibomian glands, resulting in a higher stability of the tear film, especially when treated with a combination of ciclosporin A and hydrocortisone.

Mihaltz et al., Lu et al., and Koh et al. have detected the significant improvements of the optical quality measured with wavefront aberrometry in the dry eye patients treated with topical lubricants [9,30,31]. The results of the wavefront aberromerty measurements in our study show a significantly increased optical image quality after 6 months in the eyes treated with ciclosporin A and hydrocortisone, while no significant changes could be detected in the eyes treated with ciclosporin A alone. These data may indicate long-term optical quality improvement in DED patients when treated with additional topical hydrocortisone at the beginning of a therapy with topical ciclosporin A.

Similar to Kallab et al., no significant changes of the intraocular pressure caused by hydrocortisone could be detected in our study, which demonstrates a good safety profile of the treatment with topical hydrocortisone.

However, when comparing the two treatment regimens directly to each other, no significant differences between the treatment groups could be detected, which lowers the statistical value of our findings.

Firstly, the limitation of this study was the small sample size that was partly because of the necessarily strict inclusion and exclusion criteria. Secondly, the effects of the concomitantly used lubricating eye drops could potentially have influenced our outcome data. By standardising and supplying the lubricating eye drops, we attempted to minimize this factor. A washout phase preceding the study was considered but was not implemented into the study protocol since it would be unethical to withhold adequate therapy from patients suffering from severe DED associated with Sjögren syndrome.

In conclusion, this study demonstrates the possible benefits of topical hydrocortisone additionally to topical ciclosporin A in the treatment of severe DED in patients with Sjögren syndrome. Our data indicate that if short-term hydrocortisone can safely be combined with long-term ciclosporin A therapy, it could provide the fast improvement of clinical symptoms and may have positive long-term effects on the optical image quality with the limitations of a small sample size and a lack of significant difference between the two treatment groups.

## Figures and Tables

**Table 1:** Demographic data of the study population.

Age (years)	62.3 ± 18.5

Sex (m: f)	2 : 10

Sjögren syndrome (primary: secondary)	3 : 9

**Table 2:** Study parameters at baseline compared between the two treatment regimens.

	Ciclosporin A + hydrocortisone	Ciclosporin A	*P*
*Baseline*			
Eyes	12	12	
Visual Analogue Scale	39.0 ± 20.6	35.9 ± 20.4	0.720
Tear BUT (sec)	2.8 ± 1.3	3.4 ± 1.6	0.410
Fluorescein Oxford staining grade	3.3 ± 0.5	3.1 ± 0.3	0.514
Lissamine Oxford staining grade	3.0 ± 0.9	3.0 ± 0.9	1.0
Schirmer test (mm/5 min)	3.6 ± 1.8	3.3 ± 1.4	0.590
MG dropout rate (%)	31.9 ± 12.7	33.2 ± 12.1	0.808
Corneal MTF	0.38 ± 0.11	0.39 ± 0.21	0.717
Corneal Strehl ratio	0.18 ± 0.13	0.23 ± 0.21	0.482
Intraocular pressure	14.0 ± 2.0	14.2 ± 2.7	0.932

Data are presented as mean ± SD; *P*: difference between the treatment regimens using *t* test for independent samples and Mann–Whitney U test.

**Table 3:** Study parameters at baseline and after 2 weeks and 6 months of treatment, separately for the two treatment regimens.

	Baseline	2 weeks	*P*	6 months	*P*
OSDI	56.0 ± 18.2	42.1 ± 22.0	**0.002**	28.2 ± 15.5	**<0.001**
*Ciclosporin A* *+* *hydrocortisone (CsA* *+* *Hc)*					
Visual Analogue Scale	39.0 ± 20.6	26.3 ± 19.4	**<0.001**	19.1 ± 17.7	**<0.001**
Tear BUT (sec)	2.8 ± 1.3	4.2 ± 1.3	**0.008**	5.4 ± 1.3	**0.003**
Fluorescein Oxford staining grade	3.3 ± 0.5	2.8 ± 0.7	**0.025**	1.6 ± 0.7	**0.001**
Lissamine Oxford staining grade	3.0 ± 0.9	2.7 ± 0.7	**0.046**	1.7 ± 0.8	**0.002**
Schirmer test (mm/5 min)	3.6 ± 1.8	4.6 ± 1.3	0.086	5.6 ± 1.9	**0.049**
MG dropout rate (%)	31.9 ± 12.7	—	—	30.6 ± 12.5	0.094
Corneal MTF	0.38 ± 0.11	—	—	0.50 ± 0.22	**0.037**
Corneal Strehl ratio	0.18 ± 0.13	—	—	0.30 ± 0.28	**0.042**
Intraocular pressure	14.0 ± 2.0	14.6 ± 2.0	0.289	14.4 ± 3.1	0.952

*Ciclosporin A (CsA)*					
Visual Analogue Scale	35.9 ± 20.4	24.6 ± 19.9	**0.002**	20.9 ± 18.7	**0.001**
Tear BUT (sec)	3.4 ± 1.6	3.6 ± 1.6	0.493	5.0 ± 0.9	**0.007**
Fluorescein Oxford staining grade	3.1 ± 0.3	2.9 ± 0.7	0.317	1.8 ± 0.9	**0.004**
Lissamine Oxford staining grade	3.0 ± 0.9	2.6 ± 0.7	**0.025**	1.8 ± 0.9	**0.002**
Schirmer test (mm/5 min)	3.3 ± 1.4	4.1 ± 1.3	0.057	5.6 ± 2.0	**0.050**
MG dropout rate (%)	33.2 ± 12.1	—	—	31.8 ± 12.7	0.070
Corneal MTF	0.38 ± 0.15	—	—	0.40 ± 0.16	0.923
Corneal Strehl ratio	0.22 ± 0.21	—	—	0.23 ± 0.21	0.945
Intraocular pressure (mmHg)	14.2 ± 2.7	14.5 ± 2.0	0.757	14.4 ± 2.8	0.836

Data are presented as mean ± SD; *P*: difference between baseline and values at 2 weeks and 6 months using *t* test for paired samples and Wilcoxon signed-rank test. Significant *P* values are indicated in bold.

**Table 4:** *P* values of differences in study parameters between the two treatment regimens after 2 weeks and 6 months of treatment.

	*P* (2 weeks)	*P* (6 months)
Visual Analogue Scale	0.831	0.810
Tear BUT (sec)	0.443	0.378
Fluorescein Oxford staining grade	0.799	0.755
Lissamine Oxford staining grade	0.755	0.887
Schirmer test (mm/5 min)	0.410	0.843
MG dropout rate (%)	—	0.830
Corneal MTF	—	0.205
Corneal Strehl ratio	—	0.486
Intraocular pressure	0.977	0.887

*P*: difference between the treatment regimens (ciclosporin A + hydrocortisone and ciclosporin A alone) using *t* test for independent samples and Mann–Whitney U test.

## Data Availability

The data that support the findings of this study are available from the corresponding author upon reasonable request.
